# Universality in the small scales of turbulent Taylor-Couette flow

**DOI:** 10.1126/sciadv.ady4417

**Published:** 2025-11-05

**Authors:** Julio M. Barros, Christian Butcher, Pinaki Chakraborty

**Affiliations:** Fluid Mechanics Unit, Okinawa Institute of Science and Technology Graduate University, Onna-son, Okinawa 904-0495, Japan.

## Abstract

From a humble kitchen blender to the vast galactic disks, rotating turbulent flows exhibit remarkable diversity. They are also replete with puzzling features. A notable example is the energy spectrum of a widely studied rotating flow—the turbulent Taylor-Couette (TC) flow. Previous studies have shown that, unlike other canonical turbulent flows, it does not obey Kolmogorov’s universal power law or any other power law. Here, we report measurements of the energy spectra in turbulent TC flow from unique “flying-wire” experiments where a rotating probe sweeps through the flow. In contrast with previous studies, which focused primarily on spectral power laws, we analyze spectral data collapse. Through this broader approach, we show that, contrary to the prevailing understanding, the spectral structure of small scales in turbulent TC flow is in excellent accord with the potent paradigm of Kolmogorov’s small-scale universality.

## Teaser

Rotating turbulent flows manifest a universal spectral structure.

## INTRODUCTION

Turbulent flows are no stranger to rotation. The building blocks of any turbulent flow are rotating whorls or eddies of a broad range of sizes and velocities. What distinguishes rotating turbulent flows is that the flow itself is also rotating. Does this global rotation affect the locally rotating eddies and change the building blocks of the turbulent flow?

To parse this question in concrete terms, consider the canonical rotating turbulent flow: turbulent Taylor-Couette (TC) flow. This flow is driven by and confined between two coaxial, independently rotating cylinders. Distinct from other turbulent flows, turbulent TC flow manifests several unique features. For example, in other turbulent flows, the flow attributes from the transitional state are lost as the value of the Reynolds number, *Re*, becomes large. In turbulent TC flow, however, the Taylor rolls that form during transition persist for very high values of *Re* (*1*, *2*). Similarly, even as *Re* becomes asymptotically large, turbulent TC flow exhibits multiple states instead of approaching a unique flow state (*3*). Of all its distinctive features, arguably the most confounding is its spectral structure.

The spectral structure is mathematically encapsulated in the energy spectrum. Denoted as *E*(*k*), it delineates the distribution of the kinetic energy of the turbulent eddies as a function of their size (the wave number *k* is inversely proportional to the size of an eddy). The most studied segment of *E*(*k*) is the “inertial range.” This range corresponds to 1/η≫k≫1/d , where η is the Kolmogorov length scale (the characteristic size of the smallest eddies) and *d* is the span of the radial gap between the cylinders (the characteristic size of the largest eddies). In this range, $E\left(k\right)\propto k^{\alpha }$ , where α is the spectral exponent. Kolmogorov’s phenomenological theory from 1941 (*4*) predicts α = −5/3, the celebrated “−5/3rd law.” For a given turbulent flow, the value of α is a standard diagnostic of its turbulent state. A wide range of flows (*5*), from isotropic turbulent flow (for which Kolmogorov’s theory was initially developed) to most canonical turbulent flows over walls (proviso that the spectra are measured away from the wall), conforms to Kolmogorov’s prediction, α = −5/3. A conspicuous exception is the turbulent TC flow.

Our present understanding of the unique spectral structure of turbulent TC flow has been primarily shaped by Lewis and Swinney’s seminal experiments (*6*) (hereafter, LS99). LS99, as well as subsequent studies [see, e.g., (*2*, *7*–*9*)], analyzed the streamwise component of the one-dimensional energy spectrum in the mid-gap region. In this study, we also focus on this spectrum and, for simplicity of notation, denote it as *E*(*k*), where *k* is now the streamwise wave number. For the most-studied flow configuration where only the inner cylinder rotates, studies consistently contradicted Kolmogorov’s prediction α = −5/3. Nor did they support other theoretical frameworks that might prevail in a rotating-turbulent flow, such as those predicting α = −2 or α = −3 (*10*). Instead, *E*(*k*) did not manifest a power-law region at all—α did not assume a constant value but continually varied over a broad range. The present understanding is succinctly captured in LS99’s remark: “There is no inertial range, and this flow is fundamentally different from fully developed isotropic turbulence and from turbulent pipe or channel flow, where an inertial range exists and Kolmogorov’s −5/3rd law holds at much lower Reynolds numbers.” The same theme is reflected in a recent review (*2*); among the key open problems in turbulent TC flow, they highlighted *E*(*k*), noting that the spectra “are different as compared to homogeneous isotropic turbulence, but the reason is unknown.”

The fundamentally different nature of *E*(*k*) turns even more puzzling when we consider flow configurations where both cylinders rotate. In most cases, echoing the discussion above, *E*(*k*) does not manifest a power-law region. There are, however, curious anomalies. Specifically, when the cylinders counterrotate with comparable magnitudes of angular velocities, *E*(*k*) exhibits a clear power-law region with α = −5/3 (*8*).

It is writ large that the spectral structure of turbulent TC flow exhibits an intriguing phenomenology. In most cases, it manifestly defies Kolmogorov’s theory. However, for a few select flow configurations, *E*(*k*) is clearly consonant with the theory. To wit, the spectral structure of turbulent TC flow is an enigma.

## RESULTS

### Flying-wire experiments

**Fig. 1.**
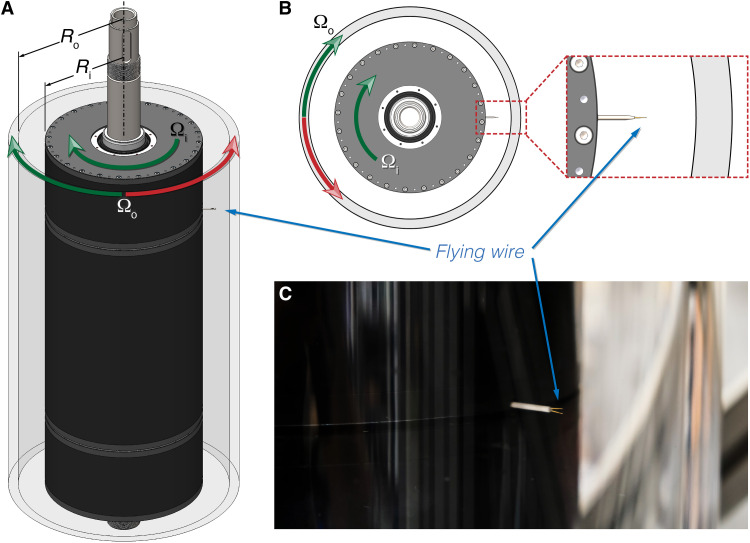
Flying-wire arrangement in OIST-TC. (**A**) Volumetric rendering of inner and outer cylinders. The flying wire is mounted on the inner cylinder (*11*). The wire measures the flow in the mid-gap region. (**B**) Schematic top view of the cylinders, with a zoomed-in view of the flying wire. (**C**) Photograph of the flying wire.

In this study, we seek to shed light on the puzzling spectral structure of turbulent TC flow. We conduct experiments using the newly built OIST-TC. This setup is discussed in detail in (*11*). Briefly, the cylinders are of length L=60.2 cm, with the inner cylinder radius $R_{i}=11.95$ cm and outer cylinder radius $R_{o}=16.0$ cm (Fig. 1A). Thus, the gap $d\equiv R_{o}-R_{i}=4.05$ cm, the radius ratio $\eta_{r}\equiv R_{i}/R_{o}=0.747$ , and the aspect ratio Γ≡L/d=14.9 . The cylinders rotate independently of each other; the working fluid is air. Unlike other canonical wall-bounded flows, TC flow is characterized by two independent Reynolds numbers: ${\text{Re}}_{i}\equiv \Omega_{i}R_{i}d/\nu$ and ${\text{Re}}_{o}\equiv \Omega_{o}R_{o}d/\nu$ , where $\Omega_{i}$ and $\Omega_{o}$ are the angular velocities of the inner and outer cylinders, respectively, and ν is the kinematic viscosity. (We use the standard convention: $\Omega_{i}\geq 0$ ). The flow can also be characterized using two other dimensionless parameters: the bulk Reynolds number ${\text{Re}}_{b}\equiv \left(\Omega_{i}\;-\;\Omega_{o}\right)\left(R_{i}\;+\;R_{o}\right)d\;/\;\left(2\nu \right)$ and the rotation ratio $a\equiv \;-\Omega_{o}\;/\;\Omega_{i}$.

**Fig. 2.**
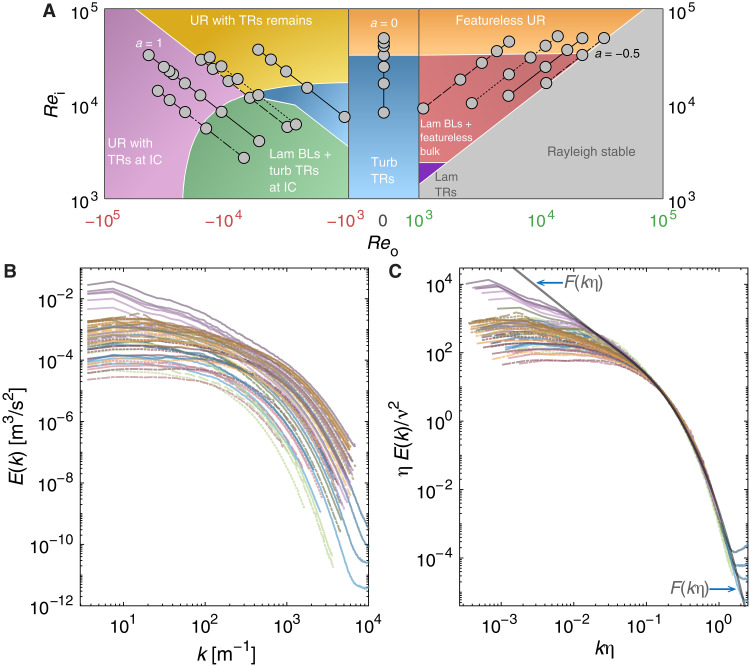
Spectral structure of turbulent TC flow. (**A**) Flow-phase diagram in the ${\text{Re}}_{o}-{\text{Re}}_{i}$ space [phase boundaries from (*20*); the value of $\eta_{r}$ in our study is similar to that in (*20*)]. The gray circles denote the $\left({\text{Re}}_{o},{\text{Re}}_{i}\right)$ points where we measure *E*(*k*). The lines join points of constant *a*, with different line types for each *a*. Abbreviations: BLs, boundary layers; IC, inner cylinder; Lam, laminar; TRs, Taylor rolls; Turb, turbulent; UR, ultimate regime. See refs. (*2*, *20*) for details of the flow phases. (**B**) Compilation of *E*(*k*) from all our experiments. In addition, for *a* = 0, we plot *E*(*k*) from stationary-wire experiments (see the Supplementary Materials). The *E*(*k*) curves are colored according to the flow-phase color in (B), with their line types corresponding to the values of *a* (see the line types in A). (**C**) *E*(*k*) curves from (B) plotted as rescaled energy spectra: $\eta E\left(k\right)/\nu^{2}$ versus kη . We also plot F(kη) [from DNS data of isotropic turbulence at ${\text{Re}}_{\lambda }=1300$ (*15*)]. Note that, at small scales, all the rescaled spectra coincide with the universal curve F(kη) , signaling Kolmogorov’s small-scale universality.

We measure *E*(*k*) in the mid-gap region [radial position = $\left(R_{i}+R_{o}\right)/2$ ] using constant-temperature-anemometry (CTA). A distinctive feature of our experiments is the “flying-wire” arrangement, wherein the CTA probe is mounted on, and rotates with, the inner cylinder, sweeping through the flow (Fig. 1). [To our knowledge, this is the first flying-wire experiment in turbulent TC flow; see also (*12*, *13*) for pioneering flying-wire experiments in transitional TC flow.] We use the flying wire to measure the azimuthal velocity time series and invoke Taylor’s frozen-turbulence hypothesis to compute *E*(*k*) from this time series (see the Supplementary Materials). The flying-wire arrangement allows us to measure *E*(*k*) with the cylinders rotating independently, affording a broad swath of the flow phase space (Fig. 2A). Furthermore, the flying-wire arrangement results in more accurate measurements of *E*(*k*) (see the Supplementary Materials).

**Fig. 3.**
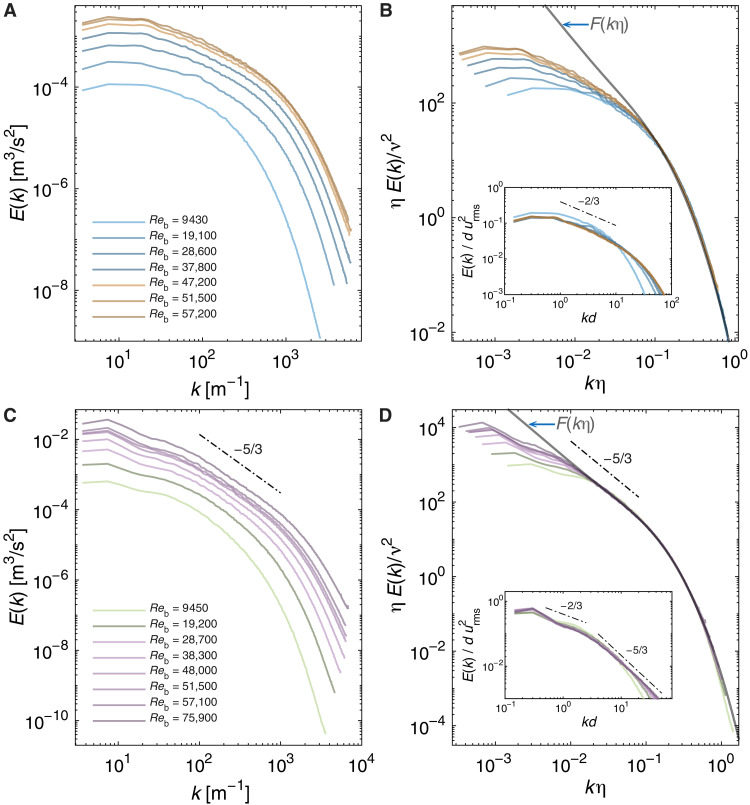
Spectral structure of two flow configurations. Spectra and rescaled spectra for the *a* = 0 (**A** and **B**) and *a* = 1 (**C** and **D**) flow configurations for different ${\text{Re}}_{b}$ values. We plot *E*(*k*) [(A) and C)] and the corresponding rescaled spectra [(B and D)]. In the insets of (B) and (D), we plot the spectra scaled using the large scales. The curves are colored as per the flow phase (see Fig. 2A).

In Fig. 2B, we plot the *E*(*k*) from all our experiments. The *E*(*k*) that correspond to the same flow phase (and are colored accordingly; see Fig. 2B) have similar shapes. Notably, most spectra betray scant evidence for α = −5/3 or any other value of the power-law exponent. The exception to this trend is the spectra for the flow configuration where the cylinders counterrotate with equal angular velocities (*a* = 1). These spectra manifest an inertial range with α = −5/3 (discussed further later; see Fig. 3C). Thus, consonant with the findings of the previous studies, our results contradict Kolmogorov’s theory for most flow configurations while confirming the theory for the *a* = 1 configuration. It would appear that our results reinforce the findings from previous studies, underscoring the puzzling nature of the spectral structure. However, parsing this data differently reveals insights, as we discuss next.

### A different approach: Data collapse

We depart from previous studies in a simple manner. Instead of focusing on the inertial range, we broaden our purview and consider *E*(*k*) over the whole extent of the small scales ( k≫1/d ). In this domain, Kolmogorov’s theory predicts (*4*)

$$E\left(k\right)=\frac{\nu^{2}}{\eta }F\left(k\eta \right)$$ (1)

where F(kη) is a flow-independent, universal function (see the Supplementary Materials for further discussion). Starting from Eq. 1 and restricting attention to the inertial range (which a subdomain of the small scales), Kolmogorov derived (*4*) $F\left(k\eta \right)\propto {\left(k\eta \right)}^{-5/3}$ , which is the −5/3rd law. That is, the −5/3rd law corresponds to the F(kη) segment that spans the inertial range. However, F(kη) has a broader realm, spanning the whole domain of the small scales.

Known as Kolmogorov’s first similarity hypothesis, Eq. 1 embodies data collapse. Specifically, it predicts that, for any turbulent flow, the rescaled energy spectrum $\eta E\left(k\right)/\nu^{2}$ attendant to the small scales collapses onto the universal curve F(kη) . This spectra collapse signals the key concept of “small-scale universality.” More generally, note that the phenomenon of data collapse is the principal significance of scaling (*14*).

To proceed, we focus on data collapse. We take each *E*(*k*) shown in Fig. 2B and plot it in the coordinates of Eq. 1, $\eta E\left(k\right)/\nu^{2}$ versus kη (Fig. 2C). (See the Supplementary Materials for calculation of η.) Whereas the plot of the original spectra (Fig. 2B) appeared to contradict Kolmogorov’s theory, the same data plotted as rescaled spectra collapse onto one master curve at high kη (small scales). The collapse is robust, irrespective of the phase space inhabited by the flow or the values of ${\text{Re}}_{i}$ and ${\text{Re}}_{o}$ (or ${\text{Re}}_{b}$ and *a*), as long as the flow is turbulent.

However, the collapse of the rescaled spectra by itself does not corroborate small-scale universality. For that, we must test whether the master curve coincides with F(kη) . Although F(kη) cannot yet be predicted theoretically, it can be readily obtained by empirical means, e.g., from Direct Numerical Simulation (DNS) data of isotropic turbulence [gray thick curve in Fig. 2C; from (*15*)]. The central figure of this paper, Fig. 2C, is now complete. Note that, in the domain of the small scales, every single rescaled spectrum from turbulent TC flow collapses onto each other—and onto F(kη) . (See the Supplementary Materials for further discussion on the collapse, including considerations of error bars.) The enigma is resolved: The spectral structure of small scales in turbulent TC flow is in excellent accord with Kolmogorov’s framework. Small-scale universality prevails.

### Spectra collapse versus power law

Why did such a simple change in approach radically transform the problem? The answer lies in Eq. 1. Although not as well known as the −5/3rd law, it is more general than the −5/3rd law. This is because, to discern the −5/3 power-law exponent, which concerns 1/η≫k≫1/d , we need a broad separation between η and *d*, which, in turn, necessitates a very large value of *Re*. By contrast, Eq. 1, which concerns k≫1/d , can hold at lower values of *Re* [see, e.g., (*16*)]. In other words, by testing for spectra collapse per Eq. 1 instead of computing the value of α, we are able to test Kolmogorov’s framework without requiring very large values of *Re*.

### Spectra collapse and the −5/3rd law

In canonical turbulent flows, the region of spectra collapse broadens with an increase in the value of *Re*, eventually encompassing the −5/3rd law at high *Re*. An examination of similar trends for turbulent TC flow is instructive. Consider, first, the most-studied flow configuration where only the inner cylinder rotates (*a* = 0). Plots of *E*(*k*) do not show an inertial range with α = −5/3 (Fig. 3A). Neither is there a clear evidence of any scaling region that might emerge with an increase in the value of ${\text{Re}}_{b}$ . However, plots of the corresponding rescaled spectra (Fig. 3B) manifest a clear trend. As we traverse the collapsed spectra curves from high to low kη (right to left along the abscissa), the first rescaled *E*(*k*) to peel off from F(kη) corresponds to the lowest value of ${\text{Re}}_{b}$ . This is followed by the second lowest value of ${\text{Re}}_{b}$ and so forth. That is, with an increase in the value of ${\text{Re}}_{b}$ , the peel-off value of kη decreases, similar to the case in canonical turbulent flows. This trend also sheds light on the onset of the −5/3rd law. For the highest ${\text{Re}}_{b}$ data in our experiments ( ${\text{Re}}_{b}\approx 57,100$ ), the rescaled *E*(*k*) peels off from F(kη) at $k\eta \approx 5\times 10^{-2}$ . However, because the F(kη) curve blends into the −5/3rd law only at $k\eta ≲10^{-1}$ , it is difficult to discern an inertial range. Thus, for the *a* = 0 flow configuration, we need spectra at higher values of ${\text{Re}}_{b}$ to clearly establish an inertial region with α = −5/3.

Now consider the flow configuration where the cylinders counterrotating with equal angular velocities (*a* = 1). Unlike the previous case, the *E*(*k*) curves show an inertial range with α = −5/3, particularly at larger values of ${\text{Re}}_{b}$ (Fig. 3C). This trend is clearer in the plot of the rescaled spectra (Fig. 3D), which shows both the collapse onto F(kη) at high kη and the broadening of the α = −5/3 region (along with lessening of the peel-off value of kη ) with an increase in the value of ${\text{Re}}_{b}$.

Analyzing the rescaled spectra with F(kη) as the reference curve highlights another aspect of the spectra beyond the purview of small-scale universality. Right after peeling off from F(kη) , the spectra at these larger scales hint at a potential scaling region (see Fig. 3, B and D). Here, the spectra not only peel off from F(kη) but also no longer collapse onto each other, indicating that their behavior is no longer dictated by Kolmogorov’s theory (*4*). To examine this region, in the insets of Fig. 3 (B and D), we plot the spectra scaled using the large scales, $E\left(k\right)/{\mathrm{du}}_{\text{rms}}^{2}$ versus *kd*, where $u_{\text{rms}}$ is the root-mean-square azimuthal velocity. Except for the ${\text{Re}}_{b}\approx 9500$ spectra, we notice a ${\left(\mathrm{kd}\right)}^{-2/3}$ scaling region, with the spectra collapsed onto one curve (see the Supplementary Materials for additional cases and further discussion). To our knowledge, a −2/3 scaling region has not been previously reported for a turbulent flow. Although suggestive, further studies are needed to examine this scaling critically.

### Trends in spectra collapse

In the previous discussion, we fixed the value of *a* and noted that the spectra collapse trends for different values of ${\text{Re}}_{b}$ were analogous to those in canonical turbulent flows. However, when we examine the trends for different values of *a*, a peculiar and puzzling aspect of turbulent TC flow becomes evident. For example, as we noted before, in the case of *a* = 0, the rescaled spectra at ${\text{Re}}_{b}\approx 57,100$ peels off from F(kη) at $k\eta \approx 5\times 10^{-2}$ , about half a decade after the onset of the inertial range (Fig. 3B). By contrast, for the case of *a* = 1, the rescaled spectra at the same value of ${\text{Re}}_{b}$ peels off from F(kη) at $k\eta \approx 10^{-2}$ , about a decade after the onset of the inertial range (Fig. 3D). Clearly, ${\text{Re}}_{b}$ cannot be used to examine the trends when the value of *a* is not fixed. The collapsed region in the rescaled spectra in Fig. 2C do not systematically broaden with an increase in the value of ${\text{Re}}_{b}$.

**Fig. 4.**
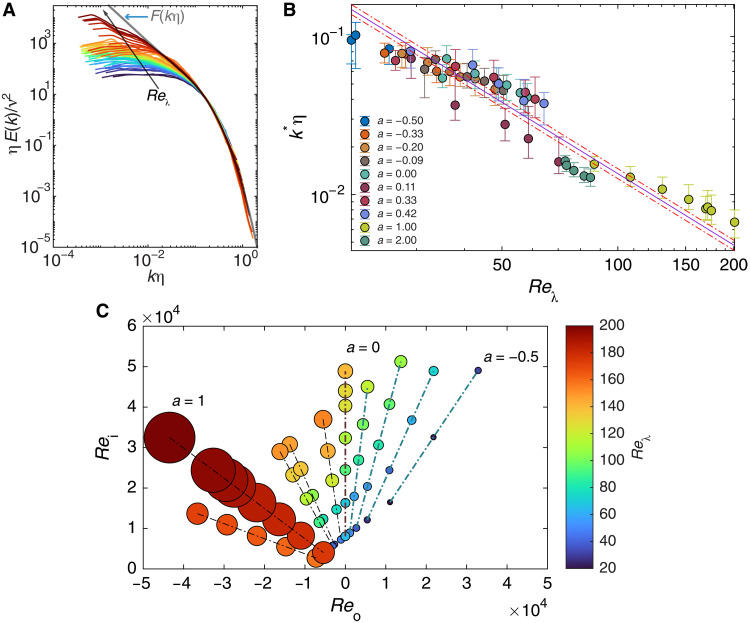
${\text{Re}}_{\lambda }$ and trends in spectra collapse. (**A**) Rescaled energy spectra, $\eta E\left(k\right)/\nu^{2}$ versus kη , of Fig. 2C, with the curves colored corresponding to the value of ${\text{Re}}_{\lambda }$ (color legend at the right of C). Note that the curves conform to the F(kη) curve for longer as ${\text{Re}}_{\lambda }$ increases. (**B**) Scaling of the dimensionless peel-off wave number, $k^{*}\eta$ , versus ${\text{Re}}_{\lambda }$ . The purple solid line is the least-squares fit to the prediction $k^{*}\eta \propto {\text{Re}}_{\lambda }^{-3/2}$ ; the red dash-dotted lines represent the 95% confidence interval of this fit. See the Supplementary Materials for a discussion of computing $k^{*}\eta$ and its attendant error bars. (**C**) ${\text{Re}}_{\lambda }$ landscape in the ${\text{Re}}_{o}-{\text{Re}}_{i}$ space. The color of the data points (filled circles) corresponds to the value of ${\text{Re}}_{\lambda }$ . For additional visual aid, we scale the circles using the value of ${\text{Re}}_{\lambda }$— the larger the value, the larger the diameter. Note that the large values of ${\text{Re}}_{\lambda }$ are localized near the *a* = 1 configuration.

This quandary can be resolved by noting that the notion of small-scale universality affords a direct link between turbulent TC flow and isotropic turbulence. In isotropic turbulence, *Re*-dependent trends are examined using the Taylor microscale Reynolds number, ${\text{Re}}_{\lambda }\equiv u_{\text{rms}}\lambda /\nu$ , where $\lambda \equiv \sqrt{15}u_{\text{rms}}\eta^{2}/\nu$ is the Taylor microscale. When examined through the lens of ${\text{Re}}_{\lambda }$ , the trends in spectra collapse become clear: Regardless of the flow configuration (as qualified by the value of *a*) or the flow phase, the region of spectra collapse monotonically broadens with an increase in the value of ${\text{Re}}_{\lambda }$ (Fig. 4A).

For a closer look at this trend, we focus on the value of kη where a given rescaled spectrum peels off from F(kη) . In Fig. 4B, we plot this value, which we denote as $k^{*}\eta$ , against ${\text{Re}}_{\lambda }$ for all the rescaled spectra. Consistent with the broadening of collapsed region discussed above, $k^{*}\eta$ decreases with an increase in ${\text{Re}}_{\lambda }$ . Moreover, we note that the data points lend credence to the theoretical prediction $k^{*}\eta \propto {\text{Re}}_{\lambda }^{-3/2}$ (see the Supplementary Materials).

By considering how ${\text{Re}}_{\lambda }$ modulates the spectra collapse, we can now understand why the spectra for the *a* = 1 flow configuration manifested an inertial range with α = −5/3, whereas the spectra for the other configurations did not, although they span comparable values of ${\text{Re}}_{b}$ . In Fig. 4C, we plot the values of ${\text{Re}}_{\lambda }$ for all our experimental data in the ${\text{Re}}_{o}‐{\text{Re}}_{i}$ space. Notably, large values of ${\text{Re}}_{\lambda }$ are localized near *a* = 1 (also see Fig. 4B). For instance, the lowest value of ${\text{Re}}_{\lambda }$ we measured in the *a* = 1 configuration ( ${\text{Re}}_{\lambda }=87$ ) is still larger than the highest value we measured in the *a* = 0 configuration ( ${\text{Re}}_{\lambda }=58$ ). Because of the high values of ${\text{Re}}_{\lambda }$ in the *a* = 1 configuration, the attendant spectra conformed to the −5/3rd law, whereas in the other flow configurations, which corresponded to smaller values of ${\text{Re}}_{\lambda }$ , the attendant spectra do not manifest an inertial range.

## DISCUSSION

In summary, unlike previous studies wherein the focus was restricted to the inertial range of *E*(*k*), we studied the spectral collapse over the entire domain of the small scales. This more general approach revealed that, contrary to the prevailing notion, the spectral structure of small scales in turbulent TC flow is not fundamentally different from other turbulent flows. Instead, it is in excellent accord with Kolmogorov’s theory.

This finding not only broadens the realm of small-scale universality, unifying turbulent TC flow with other canonical flows like isotropic turbulent flow, but it also resolves a marked inconsistency between turbulent TC flow and turbulent Rayleigh-Bénard convection. Their dynamical equations are analogous, and their many characteristic properties can be mapped onto each other (*2*, *17*). One exception, however, had been the spectral structure of small scales, for which only the turbulent Rayleigh-Bénard convection conformed with Kolmogorov’s theory (*18*). That turbulent TC flow also conforms with the theory resolves the inconsistency, strengthens the close links between turbulent TC flow and turbulent Rayleigh–Bénard convection, and underscores the remarkable reach of Kolmogorov’s simple yet powerful framework.

Last, we outline some directions for future research. In this study, we analyzed spectra collapse using the framework of Kolmogorov’s 1941 theory (*4*). Deviations from this framework constitute the rich area of small-scale intermittency (*19*). Until now, turbulent TC flow was thought to be a peculiar species of turbulence, disjointed from other canonical turbulent flows. As a result, there was little reason to probe intermittency corrections to the predictions from Kolmogorov’s theory. Our findings pave the way for investigations into the phenomenon of small-scale intermittency in turbulent TC flow.

## METHODS

See the Supplementary Materials for details of flying-wire experiments. We discuss CTA probe design, stationary-wire experiments, temperature measurements, and computing energy spectra. We also discuss probe calibration, eﬀect of temperature approximation on energy spectra, and benchmarking energy spectra.

## Supplementary Materials

### This PDF file includes:

Supplementary Discussion Figs. S1 to S8 Tables S1 and S2 References
