# Supplementary material for: Universality in the small scales of turbulent Taylor-Couette flow
Source: Sci Adv. 2025 Nov 5;11(45):eady4417. doi: 10.1126/sciadv.ady4417 (PMC12588277; doi:10.1126/sciadv.ady4417)
Supplement: Supplementary file 1 — Supplementary Discussion Figs. S1 to S8 Tables S1 and S2 References [file sciadv.ady4417_sm.pdf]

Science Advances
AAAS

Supplementary Materials for

Universality in the small scales of turbulent Taylor-Couette flow

Julio M. Barros *et al.*

Corresponding author: Pinaki Chakraborty, pinaki@oist.jp

Sci. Adv. **11**, eady4417 (2025)
DOI: 10.1126/sciadv.ady4417

This PDF file includes:

Supplementary Discussion
Figs. S1 to S8
Tables S1 and S2
References

1 Flying-wire experiments

A unique feature of the OIST-TC setup is the flying-wire arrangement for constant-temperature-anemometry (CTA). In this arrangement, the CTA probe (the "flying wire") samples the flow in the mid-gap region (radial position $R_m = (R_i + R_o)/2$) whilst rotating with the inner cylinder. The main advantage of the flying-wire arrangement is that it allows us to measure the flow velocity whilst either or both cylinders are rotating. An additional advantage is that because the flying wire rotates faster than the local fluid, the mean velocity measured by the wire is higher than the mean local flow velocity. This allows us to use Taylor's frozen-turbulence hypothesis without needing a correction for turbulence intensity (see Sec. 3.1).

CTA probe: We custom designed a CTA probe (manufactured by Dantec) based on the specifications discussed in [21]. This design allows us to accurately resolve the small scales of the flow and avoid contamination from non-longitudinal wavenumbers [22, 23]. The probe is made of Tungsten wire of diameter $d_{hw} = 2.5\mu$m and sensing length $l = 0.5$ mm. In Table S-1, we report the range of spatial and temporal resolution for the probe. Additional details of the probe (placement, electrical circuit, and CTA settings) are discussed in [11].

η [μm]	η/l	k_{max} [m^{-1}]	$k_{max}\eta$	$f_e\eta^2/\nu$
90.4–568	0.18–1.14	748–6826	0.30–1.54	52.4–2058.8

Table S-1: CTA probe parameters (minimum and maximum values) for flying-wire experiments. Here, η is the Kolmogorov length scale, k_{max} is the maximum resolved wavenumber of $E(k)$, and f_e is the effective data-sampling frequency. We estimated k_{max} from the inflection point of $k^2E(k)$ [11, 16]. To estimate f_e, we first conduct a square-wave test of the CTA probe using slightly over-damped settings [21]. This yielded a cut-off frequency of ~ 120 kHz for a 10 m/s flow. Based on this result, we set $f_e = 100$ kHz (for which we sampled the CTA voltage signal at 200 kHz and applied a 100 kHz low-pass filtering to avoid aliasing [11]).

Stationary-wire experiments: In addition to the flying-wire experiments, we carried out CTA experiments in the standard arrangement where the probe is stationary. The probe support is introduced into the flow via a small hole in one of the acrylic windows on the top flange [11]. The probe is located as close as possible to the axial and radial position of the probe in the flying-wire experiments. Because the probe is introduced through the top flange, stationary-wire experiments can be carried out when only the inner cylinder is rotating (and the outer cylinder is at rest), i.e., for $a = 0$.

Temperature: The temperature was measured via Platinum Resistance Temperature Detectors (RTDs) glued to the inner wall of the inner cylinder's middle section [11]. The temperature in all experiments ranged between 23°C and 25°C; for a single experiment, the maximum temperature variation was < 0.1°C. In flying-wire experiments, we approximate the flow temperature using the RTD sensor located closest in the axial direction to the CTA probe. In stationary-wire experiments, we directly used the temperature measured by the CTA temperature probe located right adjacent to (and in tandem with) the CTA velocity probe.

Energy spectra: We compute $E(k)$ from CTA velocity time series. For all measurements, the acquisition duration was set as $t_{acq} = 54,200 \times t_{eddy}$, where $t_{eddy} = \frac{d}{(\Omega_i - \Omega_o)R_m}$ is the eddy turn-over time. We transform the velocity time series to velocity spatial series using Taylor's frozen-turbulence hy-

pothesis (cf. Sec 3.1) and compute $E(k)$ from the velocity spatial series using the Welch periodogram. In this method, the velocity spatial signal is subdivided into N segments. With an overlap of 50% between segments, we get $2N$ periodograms. Then, we compute the energy spectrum for each periodogram using the discrete Fourier transform. The average of these spectra is the $E(k)$ and its standard error (standard deviation normalized by $\sqrt{2N}$) is the statistical uncertainty. (In our experiments, the average value of $2N$ is 1500; we chose this value to set the low-wavenumber cut-off as $k_{\min}d = 0.15$.)

1.1 Calibration

The CTA probe is calibrated using the Dantec StreamLine Pro Automatic calibrator which produces a controlled jet flow for calibration [11]. In-situ calibration, although ideal, was not possible because the mid-gap flow velocity is not known *a priori*. The velocity range for the calibration spanned from 0.5 m/s to 30 m/s. The calibration was performed before installing the probe in the OIST-TC setup. To check for any potential calibration drift, an additional calibration was performed after each measurement campaign, and the results were compared with the original calibration. In Fig. S-1, we show representative data from the original and post-measurements calibrations. We note that there is no discernible drift in the calibration data.

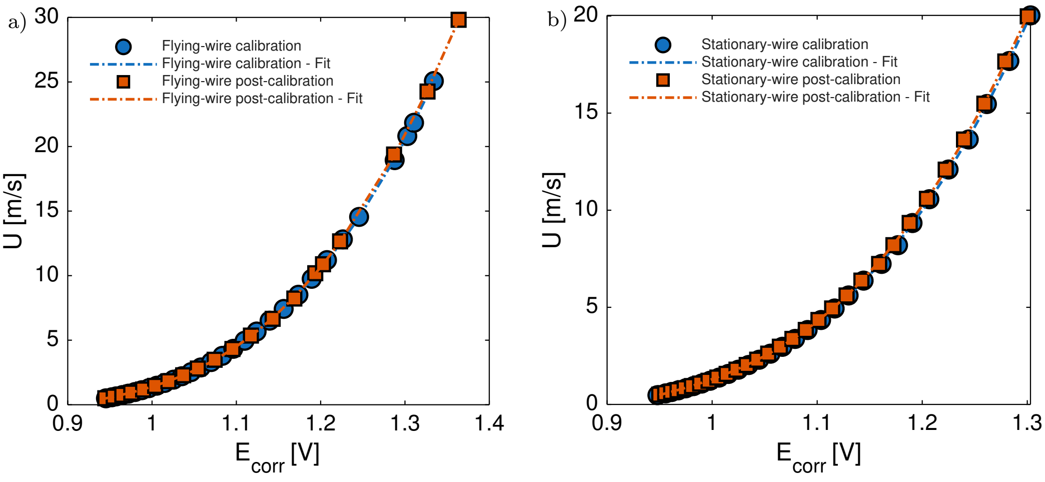

Figure S-1: Calibration data from original (blue) and post-measurements (red) calibrations for a) flying-wire experiments and b) stationary-wire experiments. The calibration data are plotted as flow velocity, U, vs. corrected CTA voltage signal, E_{corr}. We also plot calibration fits, for which we use 4th-order polynomials.

An additional consideration is to correct the CTA voltage signal due to temperature difference between the calibration temperature and measurement temperature. For that, we use Bearnman's method [24, 25]:

$$E_{\text{corr}} = \left(\frac{T_w - T_0}{T_w - T_a}\right)^{\alpha} . E_a, \tag{S-1}$$

where E_{corr} is the corrected voltage signal, T_w is the hot-wire hot temperature, T_0 is the reference temperature taken during the calibration, T_a is the ambient temperature during measurements, α is the correction exponent, and E_a is the acquired voltage signal. We use $\alpha = 0.3$ to expand the

temperature change range [25]. We used a standard 4th-order polynomial fit on the calibration data points [26], which was then used to convert the voltage signal from the CTA probes to velocity time signal.

1.2 Testing the effect of temperature approximation on energy spectra

As noted before, in the flying-wire experiments, the mid-gap flow temperature was not directly measured. Instead, it was approximated as the value measured by the closest RTD sensor, T_a. This temperature was then used to compute the fluid kinematic viscosity and to correct the CTA voltage signal for the temperature difference with the calibration temperature, T_0 (cf. Eq. (S-1)). However, this temperature may be different from the temperature of the mid-gap flow. Here we test the influence of approximating the mid-gap flow temperature on the energy spectrum.

We begin by quantifying the temperature difference using the results of stationary-wire experiments. As noted before, in these experiments, we directly measure the mid-gap flow temperature using the CTA temperature probe. We compare this temperature with that from closest RTD sensor (Fig. S-2). Across all experiments, the measured value from the CTA temperature probe was lower than T_a; the average temperature difference was $\sim 0.15°$ C, and the maximum temperature difference was $\sim 0.25°$ C.

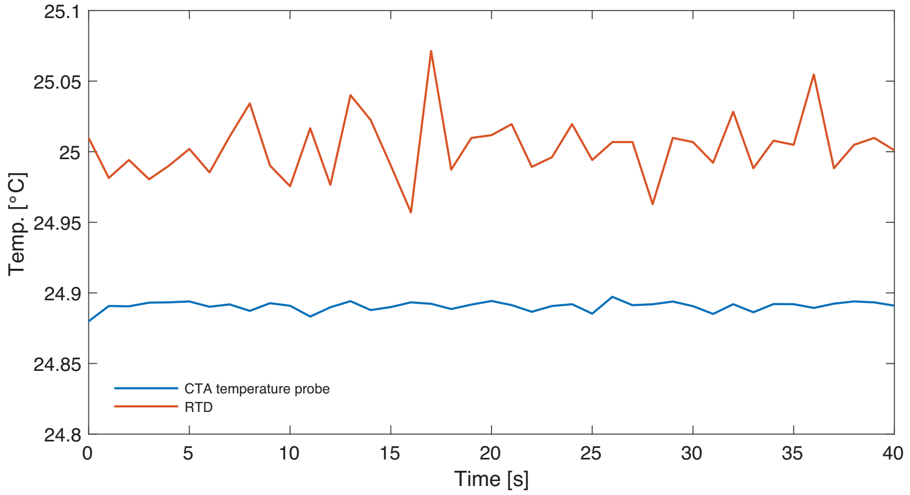

Figure S-2: A representative time series of temperature measured by the CTA temperature probe (blue) and the closest RTD sensor, T_a (orange). In this experiment, only the inner cylinder was rotating ($\Omega_i/2\pi = 16.67$ s^{-1}).

Now we test the effect of the temperature difference on the energy spectrum. For the flying-wire experiments, we computed the energy spectra corresponding to different temperatures: for baseline, we used T_a, and, for comparison, we changed this temperature by $\pm 0.5°$C (twice the maximum temperature difference noted above). For both the dimensional $E(k)$ and its attendant rescaled version, we found that the temperature difference has no discernible effect (Fig. S-3). Analogous tests using data from stationary-wire experiments yielded the same result. We conclude that approximating the mid-gap flow temperature using the closest RTD probe has a negligible effect on the energy spectrum.

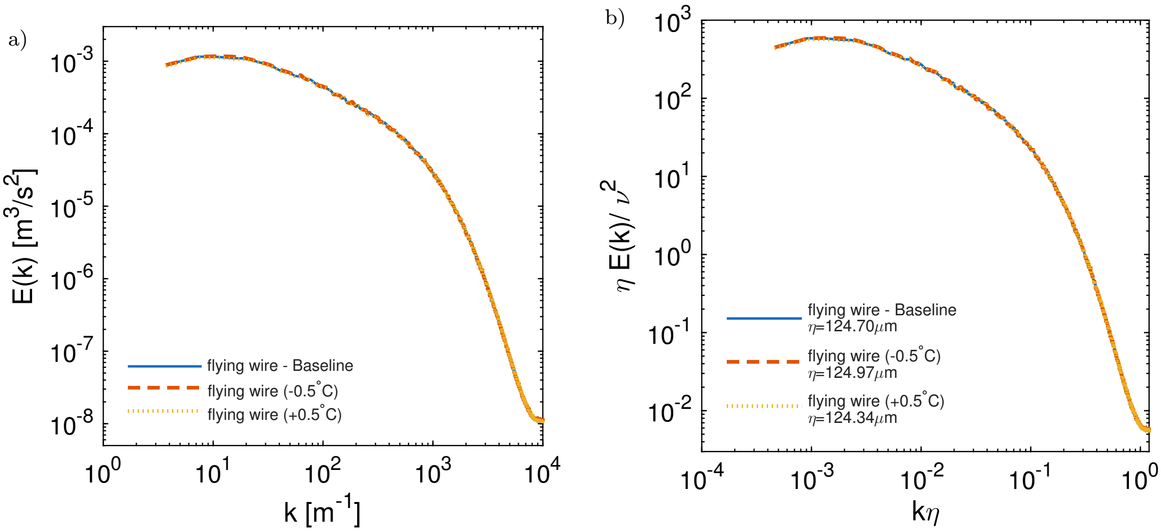

Figure S-3: Energy spectra, dimensional (a) and rescaled (b), with temperature corrections corresponding to T_a (blue solid line; baseline), $T_a + 0.5°$ C (orange dashed line), and $T_a - 0.5°$ C (yellow dotted line). In this experiment, only the inner cylinder was rotating at 1000 rpm.

1.3 Benchmarking

We used Laser Doppler Velocimetry (LDV) and Direct Numerical Simulations (DNS) to benchmark the energy spectra from flying-wire experiments. The results from LDV are discussed in [11] (see Sec. 5.2). The results from DNS are discussed in Sec. 3 (see Figs. S-4 and S-6). For both LDV and DNS benchmarking tests, we note that the flying-wire spectra are in excellent accord with their counterpart from LDV or DNS.

2 A note on Kolmogorov's first similarity hypothesis

The leitmotif of our analysis is the spectra collapse dictated by Kolmogorov's first similarity hypothesis (Eq. 1 in the manuscript):

$$E(k) = \frac{\nu^2}{\eta} F(k\eta). \tag{S-2}$$

This equation is predicated on dimensional analysis. Kolmogorov's key insight was to argue that in the domain of small scales, the energy spectrum only depends on k, ν, and ε, where ε is the mean energy dissipation rate per unit mass [4]. With this functional dependence, applying the Buckingham Pi theorem [27] yields

$$\frac{E(k)}{\varepsilon^{1/4}\nu^{5/4}} = \Phi\left(k\frac{\nu^{3/4}}{\varepsilon^{1/4}}\right), \tag{S-3}$$

where Φ is a dimensionless function. Introducing the Kolmogorov length-scale, $\eta \equiv \nu^{3/4}/\varepsilon^{1/4}$, the above relation can be expressed as:

$$\frac{\eta E(k)}{\nu^2} = \Phi(k\eta), \tag{S-4}$$

which is Eq. (S-2), wherein we also note that $\Phi(k\eta) = F(k\eta)$.

Now consider a different starting point. Instead of the classical choice of functional dependence (k, ν, and ε), we can argue that in the domain of small scales, the energy spectrum only depends on k, ν, and η. Now, the applying the Buckingham Pi theorem yields:

$$\frac{\eta E(k)}{\nu^2} = \tilde{\Phi}(k\eta), \tag{S-5}$$

where $\tilde{\Phi}$ is a dimensionless function. Comparing with Eq. (S-4), we note that $\tilde{\Phi}(k\eta) = \Phi(k\eta)$. While, in general, a different choice of functional dependence yields different functional forms (which can be transformed into each other; see, e.g., [27]), in this particular case, it yields exactly the same functional form.

3 Tests using Direct Numerical Simulations

In the manuscript, we used Taylor's frozen-turbulence hypothesis to compute $E(k)$ and Taylor's isotropic formula to compute η. We test their validity in turbulent TC flow using DNS. Additionally, as noted in the previous section, we use the DNS energy spectra to benchmark the flying-wire energy spectra.

We used a DNS code for TC flow developed by Dr. Ashley P. Willis based on the Navier–Stokes solver Openpipeflow [28]. In the radial direction, r, it uses a Chebyshev grid and computes the spatial derivatives using finite differences. In the azimuthal (θ) and axial (z) directions, it uses a uniformly-spaced grid and computes spatial derivatives using Fourier series.

We simulated two cases: $a = 0$ (inner cylinder rotation only) and $a = 1$ (cylinders counter-rotating with equal angular velocities). The Reynolds numbers for the two cases were picked to match corresponding cases from our flying-wire experiments. The domain sizes in the axial and azimuthal directions were $L_z = \pi d$ and θ-span $= \pi$ radians, respectively. The radius ratio, η_r, was set to match that of the OIST-TC setup. The time step was dynamically adapted based on the Courant number, whose value was set to 0.5. We ran the simulations for $\sim 9,700$ advection time units (U_i/d), where $U_i = \Omega_i R_i$ is the tangential velocity of the inner cylinder, and computed the flow properties using the flow fields from the last ~ 750 advection time units. Similar to the experiments, we restrict attention to the mid-gap region ($r = R_m$). In Table S-2, we list the simulation parameters.

Table S-2: DNS parameters.

$\mathrm{Re_i}$	$\mathrm{Re_o}$	$\mathrm{Re_b}$	$a = -\Omega_o/\Omega_i$	Grid size $[z \times \theta \times r]$	η_r	L_z	θ-span
8000	0	9355	0	$160 \times 512 \times 128$	0.747	πd	π
4040	-5409	9500	1	$160 \times 512 \times 128$	0.747	πd	π

3.1 Taylor's frozen-turbulence hypothesis

Although previous studies have used Taylor's frozen-turbulence hypothesis to compute $E(k)$ in turbulent TC flow (see, e.g., [6, 29]), to our knowledge, its validity has not been previously tested in turbulent TC flow. We test Taylor's hypothesis for flows with $a = 0$ and $a = 1$. For both cases, we compute $E(k)$ from flying-wire experiments (where we invoke Taylor's hypothesis) and

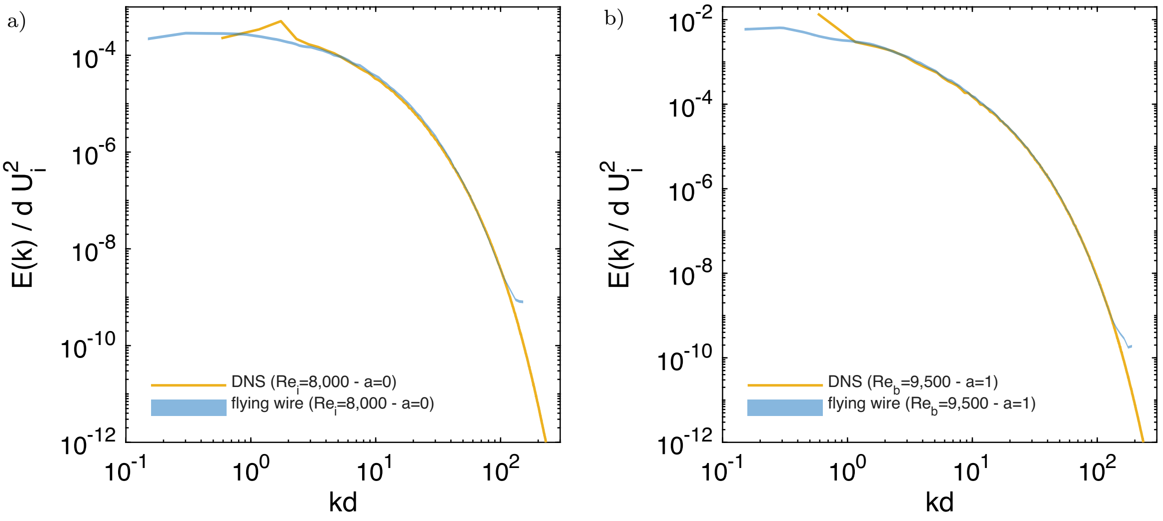

Figure S-4: Comparing $E(k)$ between flying-wire (blue) and DNS (yellow) results for: (a) $\mathrm{Re_i} = 8,000$ and $a = 0$ and (b) $\mathrm{Re_b} = 9,500$ and $a = 1$. The spectra are normalized using U_i and d as the velocity and length scales, respectively. For the flying-wire spectra, we plot a band whose thickness at a fixed value of kd corresponds to 95% of the confidence interval computed from the statistical uncertainty in $E(k)$ (see Sec. 1) normalized by $d\,U_i^2$. (The band is centered on the average value of $E(k)$ normalized by $d\,U_i^2$.) We note that the uncertainty is small. Also note that the DNS $E(k)$ show a peak at the large scales ($kd \approx 1$). This feature has been noted before and attributed to the periodicity imposed in the computational domain (see Fig. 8 in [33]). Consistent with this explanation, no corresponding peak is present in the flying-wire $E(k)$.

from DNS (where we use direct computation). For the experiments, we first use Taylor's hypothesis to transform the measured velocity time signal to velocity spatial signal using the time-averaged velocity as the convection velocity [30]. Then, we compute $E(k)$ from the velocity spatial signal. For the DNS, we compute $E(k)$ directly from the (spatial) Fourier modes. In Fig. S-4, we compare the $E(k)$ from experiments and DNS. The excellent accord between the spectra, particularly at the small scales, indicates that Taylor's hypothesis provides a reliable tool for computing $E(k)$ from velocity time signal.

The flying-wire experiments are particularly attractive for invoking Taylor's hypothesis. It is known that Taylor's hypothesis can be applied without any correction when the turbulent intensity, TI (the ratio of r.m.s. of velocity fluctuations and the mean velocity) is less than about 10% [31, 32]. As noted in Sec. 1, in the flying-wire arrangement, because the mean velocity measured by the moving CTA probe is higher than the local flow velocity, the value of TI is reduced. Indeed, the highest TI measured in all runs for the flying-wire experiments was below 10%, indicating that Taylor's hypothesis can be applied without any correction for TI.

3.2 Taylor's isotropic formula

The Kolmogorov length-scale η can be computed using its definition:

$$\eta \equiv \left(\frac{\nu^3}{\varepsilon}\right)^{1/4}. \tag{S-6}$$

Computing ε is particularly challenging for it involves 12 gradient moments:

$$\varepsilon = 2\nu\langle \boldsymbol{\nabla u}' \dot{:} \boldsymbol{\nabla u}' \rangle, \tag{S-7}$$

where $\boldsymbol{u}'$ is the fluctuating velocity field and $\langle\rangle$ denotes an ensemble average. Further, these moments of velocity gradients are largely determined by the flow variation in the small length-scales. Computing them accurately thus entails a high spatial resolution of the velocity field. As a result, computing η using Eqs. (S-6) and (S-7) is a difficult task, particularly using experimental data.

For isotropic turbulence, Taylor [34] transformed Eq. (S-7) into a remarkably simple formula:

$$\varepsilon_{iso} = 15\nu \int_0^\infty k^2 E(k) dk, \tag{S-8}$$

where ε_{iso} represents ε in isotropic turbulence and recall that in the notation of the manuscript, $E(k)$ represents the streamwise component of the energy spectrum (with k as the streamwise wavenumber). In contrast with Eq. (S-7), Eq. (S-8) provides a relatively straightforward way to estimate ε. Indeed, most experiments use Eq. (S-8) to estimate ε. Recently, it has been shown [35] that invoking rotational symmetry or reflectional symmetry or both leads to Taylor's isotropic formula (Eq. (S-8)). More generally, because the value of ε_{iso} (or ε) is largely determined by the small scales, Taylor's isotropic formula furnishes a good approximation for flows with local isotropy. These include turbulent wall-bounded flows (with the condition that the spatial location is not near the wall [36, 37]) such as pipe flow [16, 38], boundary-layer flow [5], and TC flow [6, 29].

Of particular relevance to our analysis is to test the effect of Eq. (S-8) on the data collapse ($\eta E(k)/\nu^2$ vs. $k\eta$) analyzed in the manuscript. Specifically, we are interested in how η computed as $\eta = (\nu^3/\varepsilon)^{1/4}$ (cf. Eqs. (S-6) and (S-7)) and $\eta = (\nu^3/\varepsilon_{iso})^{1/4}$ (cf. Eqs. (S-6) and (S-8)) may affect the data collapse.

For flows with $a = 0$ and $a = 1$, we carry out two tests. In the first test, using the DNS data, we compute η using two approaches ($\eta = (\nu^3/\varepsilon)^{1/4}$ and $\eta = (\nu^3/\varepsilon_{iso})^{1/4}$) and plot the corresponding rescaled energy spectra, $\eta E(k)/\nu^2$ vs. $k\eta$ (Fig. S-5). In the second test, we compute the rescaled energy spectra from flying-wire experiments (where we use $\eta = (\nu^3/\varepsilon_{iso})^{1/4}$) and from DNS (where we use $\eta = (\nu^3/\varepsilon)^{1/4}$) (Fig. S-6). In both tests and for both cases ($a = 0$ and $a = 1$), the excellent accord between the rescaled energy spectra indicates that estimating η using Taylor's isotropic formula provides a reliable test of the data collapse.

4 Additional discussion of the $-2/3^{\mathrm{rd}}$ scaling

In the manuscript, we discussed the $(kd)^{-2/3}$ scaling region for the flow configurations of $a = 0$ and $a = 1$. Here we analyze the spectra from all our experiments. In Fig. S-7, we plot all the cases where two criteria are met: (i) the scaling region spans more than half a decade in kd and (ii) for a given flow configuration (fixed value of a), criterion (i) is satisfied for spectra corresponding to at least two values of $\mathrm{Re_b}$. We plot the spectra scaled using the large scales: $E(k)/du_{\mathrm{rms}}^2$ vs. kd. We identify the

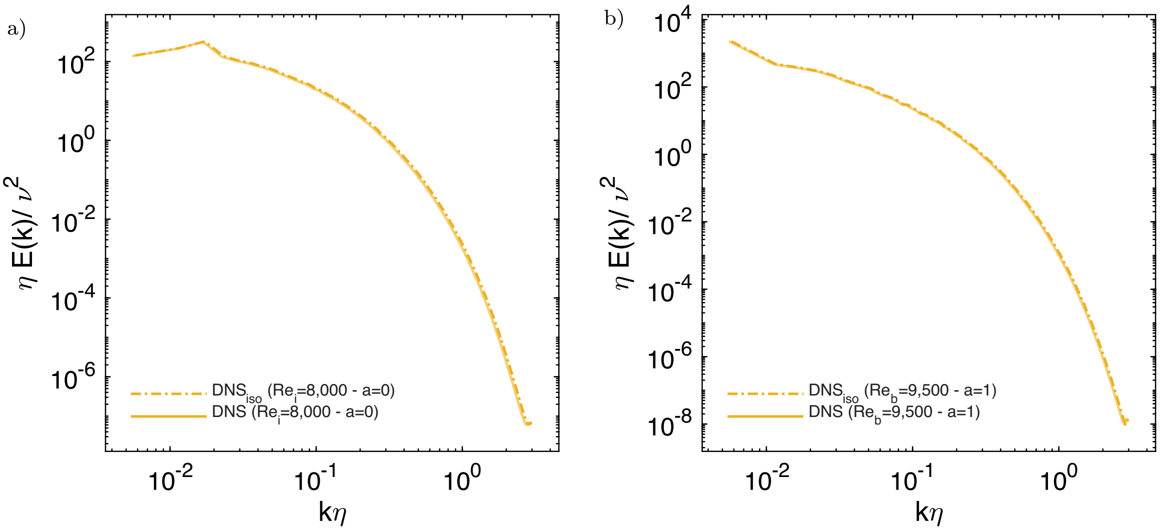

Figure S-5: Comparing rescaled energy spectra, $\eta E(k)/\nu^2$ vs. $k\eta$, using DNS results for: (a) $\mathrm{Re_i} = 8,000$ and $a = 0$ and (b) $\mathrm{Re_b} = 9,500$ and $a = 1$. We compute η using two approaches: $(\nu^3/\varepsilon)^{1/4}$ (DNS; solid line) and $(\nu^3/\varepsilon_{iso})^{1/4}$ ($\mathrm{DNS_{iso}}$; dash-dotted line).

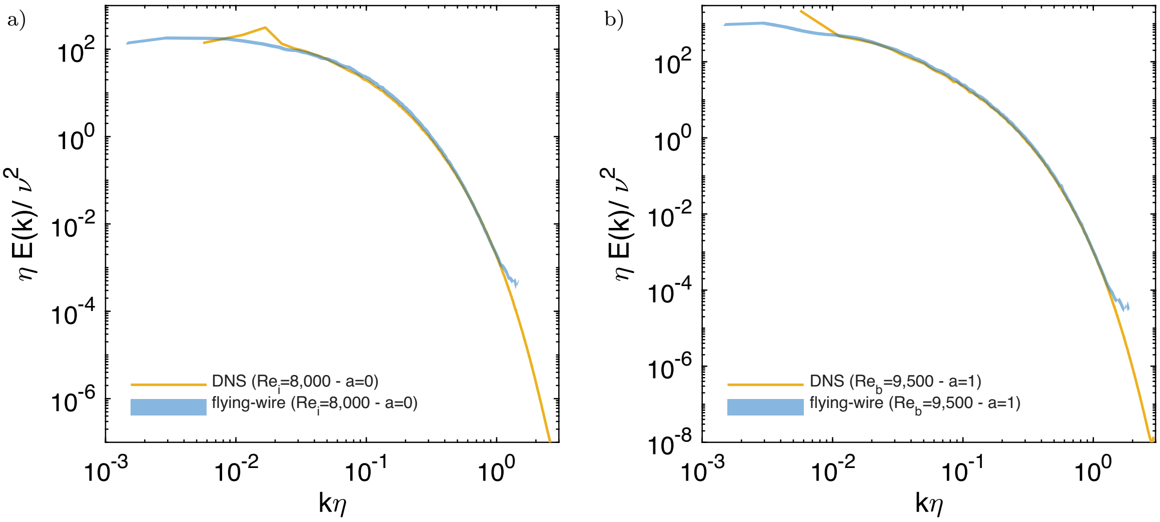

Figure S-6: Comparing rescaled energy spectra, $\eta E(k)/\nu^2$ vs. $k\eta$, between flying-wire (blue) and DNS (yellow) results for: (a) $\mathrm{Re_i} = 8,000$ and $a = 0$ and (b) $\mathrm{Re_b} = 9,500$ and $a = 1$. For DNS data, we compute $E(k)$ and η without invoking any assumptions. For the flying-wire data, we compute $E(k)$ using Taylor's hypothesis and η using Taylor's isotropic formula. Additionally, for the flying-wire data, we plot a band whose thickness at a fixed value of $k\eta$ corresponds to the combined effect of the statistical uncertainty in $E(k)$ (cf. Fig. S-4) and the attendant uncertainty in η. For the latter, we compute η using the upper and lower boundaries of the $E(k)$ band (cf. Fig. S-4). We note that the uncertainty is small.

scaling region as the wavenumber range wherein the coefficient of determination, R^2, for the least-squares fit to the $(kd)^{-2/3}$ scaling satisfies $R^2 \geq 0.98$. Except for $a = 1$, the scaling region spans about one decade, $10^0 \lesssim kd \lesssim 10^1$. Further, for $\mathrm{Re_b} \gtrsim 2 \times 10^4$, the spectra for the different values of $\mathrm{Re_b}$ collapse onto one curve in the scaling region. However, the necessary conditions to realize this scaling are unclear. For instance, whereas the value of Re_λ modulates the $-5/3$ scaling region, it appears to have little purchase on setting the $-2/3$ scaling region. Indeed, while the spectra for the $a = 1$ flow configuration manifest the clearest $-5/3$ scaling region of all our experiments, they have the shortest $-2/3$ scaling region among the cases shown in Fig. S-7.

5 Dimensionless peel-off wavenumber, $k^*\eta$

5.1 $k^*\eta$ scaling

We derive a scaling relation for $k^*\eta$ scaling based on two key assumptions. For the first assumption, we posit:

$$k^* \propto 1/L, \tag{S-9}$$

where L is a characteristic large scale [16]. In this analysis, we leave L unspecified (later we shall see that it cancels out of the $k^*\eta$ scaling). From Eq. (S-9) we get:

$$k^*\eta \propto \frac{\eta}{L}. \tag{S-10}$$

We now turn attention to η/L.

For the second assumption, we invoke the "dissipation anomaly" [34]:

$$\varepsilon \propto \frac{u_{\mathrm{rms}}^3}{L}. \tag{S-11}$$

Using $\eta \equiv \nu^{3/4}/\varepsilon^{1/4}$, the above equation can be expressed as:

$$\frac{\eta}{L} \propto \left(\frac{u_{\mathrm{rms}} L}{\nu}\right)^{-3/4}. \tag{S-12}$$

Noting that $\lambda \propto u_{\mathrm{rms}}\eta^2/\nu$ and $\mathrm{Re}_\lambda \equiv u_{\mathrm{rms}}\lambda/\nu$, Eq. (S-12) transforms to:

$$\frac{\eta}{L} \propto \mathrm{Re}_\lambda^{-3/2}. \tag{S-13}$$

Combining with Eq.(S-10) yields the scaling relation:

$$k^*\eta \propto \mathrm{Re}_\lambda^{-3/2}. \tag{S-14}$$

5.2 Computing $k^*\eta$

To compute $k^*\eta$, we first calculate the ratio between $F(k\eta)$ and a given rescaled spectrum, $\eta E(k)/\nu^2$. In the region of spectra collapse, this ratio is ≈ 1, whereas at larger scales (at low values of $k\eta$), it assumes values as large as 10^2 (Fig. S-8). We compute $k^*\eta$ as the lowest value of $k\eta$ where the ratio

becomes ≈ 1. To calculate the error bar for $k^*\eta$, we repeat this process using the upper and lower boundaries of the uncertainty band of the rescaled spectrum (cf. Fig. S-6).

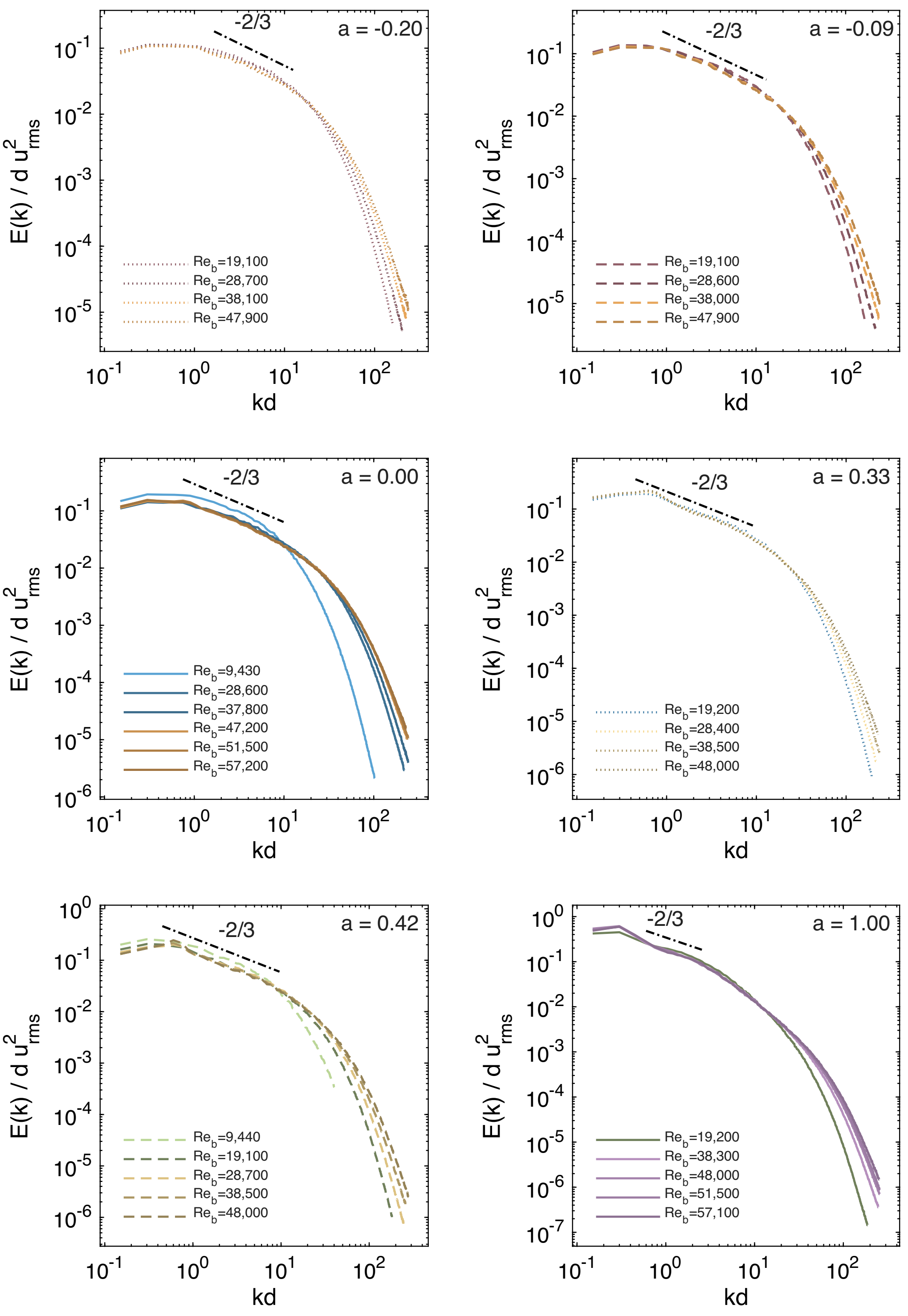

Figure S-7: The $(kd)^{-2/3}$ scaling. We plot the spectra scaled as $E(k)/du_{\rm rms}^2$ vs. kd. Different panels correspond to different values of a. The wavenumber span of the $-2/3$ slope line (black dash-dotted line) corresponds to the average extent of the scaling region for the spectra plotted in the panel.

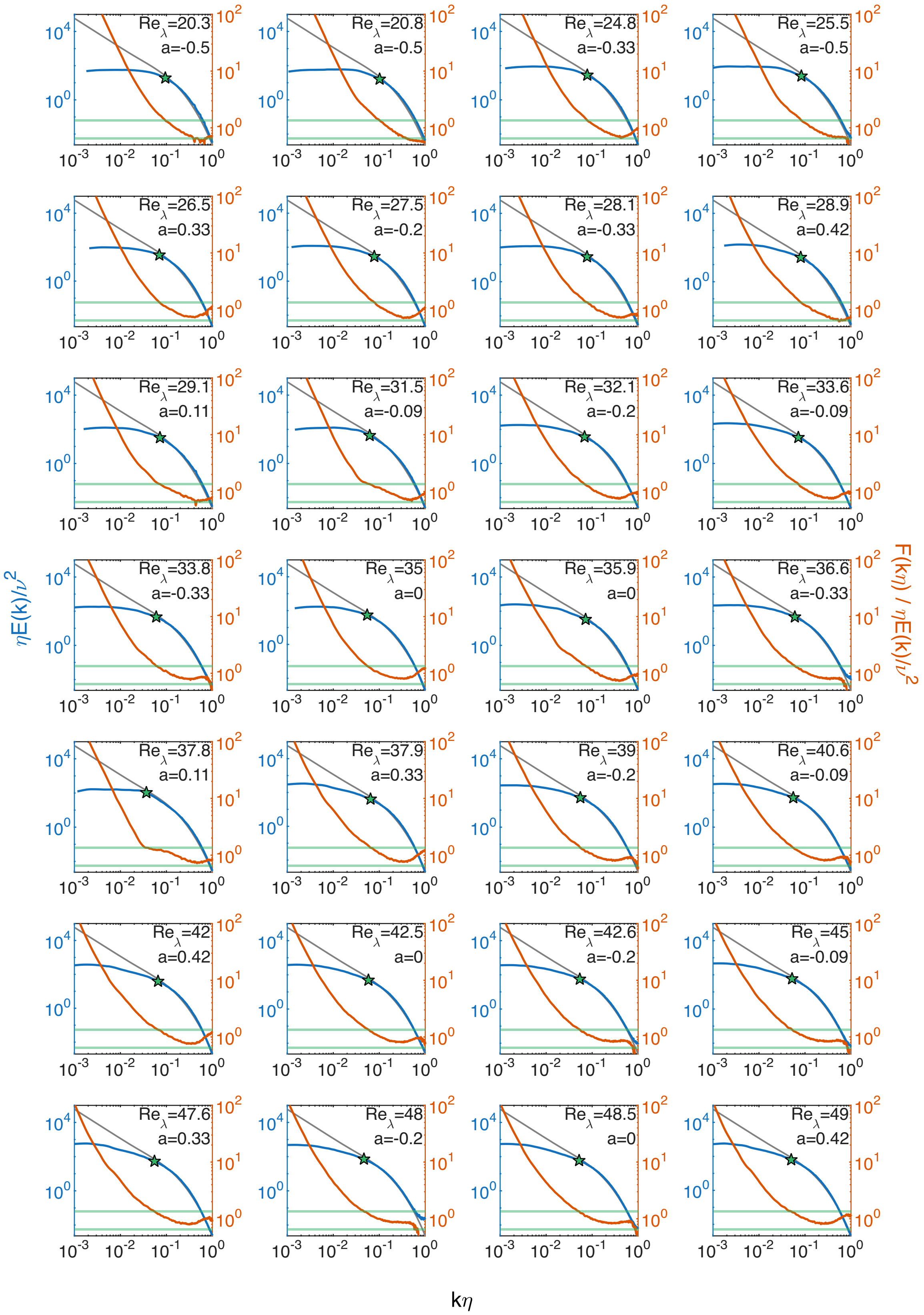

Figure S-8: (Figure continued on the next page) Computing $k^*\eta$. The panels are arranged by increasing value of Re_λ. In each panel, we plot $F(k\eta)$ (grey), $\eta E(k)/\nu^2$ (blue), and their ratio (red) as a function of $k\eta$. The position of $k^*\eta$ is marked with a star; it corresponds to the lowest $k\eta$ value at which the ratio value falls between 1 ± 0.35 (marked by two cyan lines). (The results are comparable for thresholds of ± 0.25 and ± 0.5.)

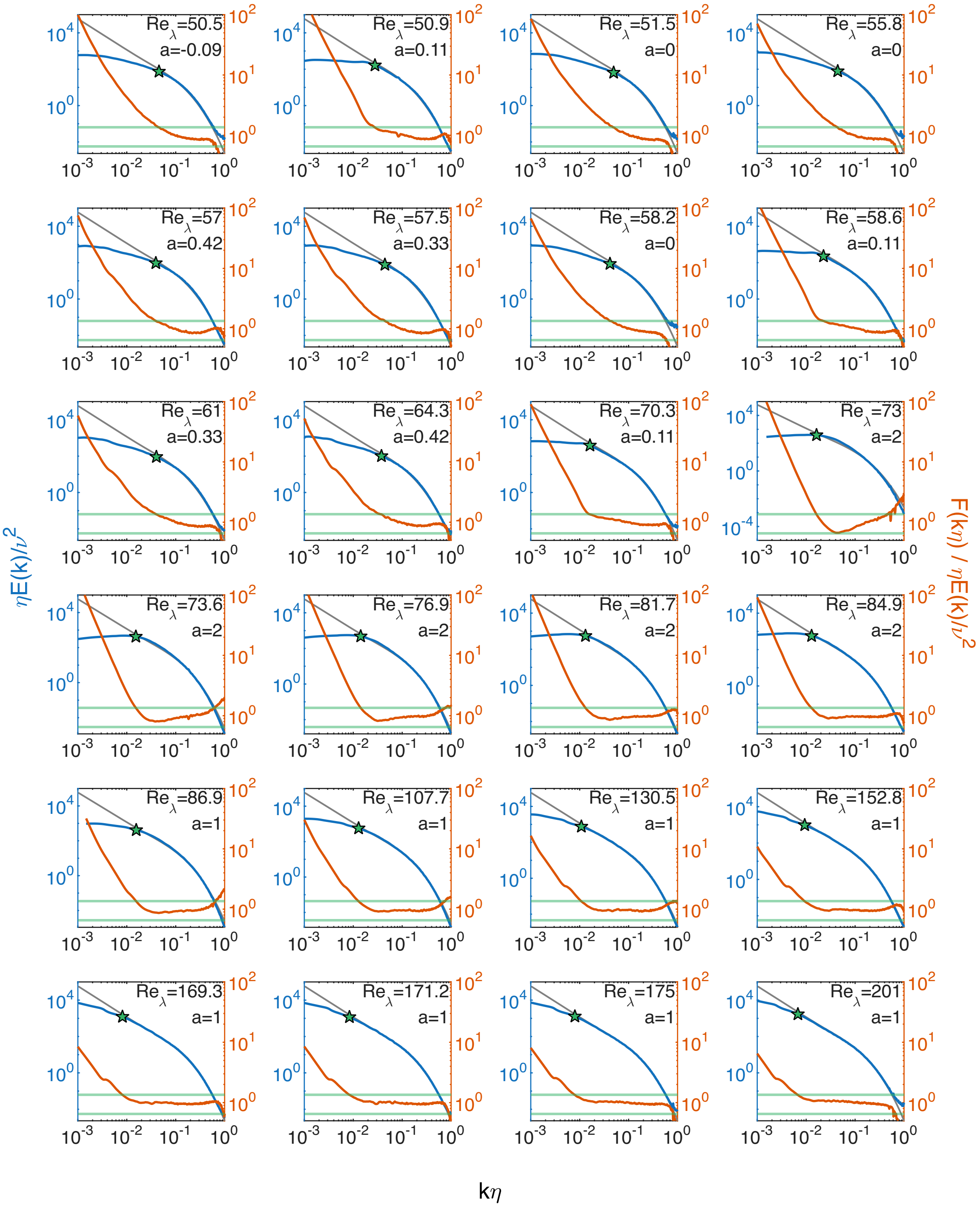

Figure S-8: (Figure continued from the last page) Computing $k^{*}\eta$.

REFERENCES AND NOTES

1. C. D. Andereck, S. S. Liu, H. L. Swinney, Flow regimes in a circular couette system with independently rotating cylinders. *J. Fluid Mech.* **164**, 155–183 (1986).

2. S. Grossmann, D. Lohse, C. Sun, High-Reynolds number Taylor–Couette turbulence. *Ann. Rev. Fluid Mech.* **48**, 53–80 (2016).

3. S. G. Huisman, R. C. A. Van Der Veen, C. Sun, D. Lohse, Multiple states in highly turbulent Taylor–Couette flow. *Nat. Commun.* **5**, 3820 (2014).

4. A. N. Kolmogorov, The local structure of turbulence in incompressible viscous fluid for very large Reynolds numbers. *Dokl. Akad. Nauk SSSR* **30**, 301–305 (1941).

5. S. G. Saddoughi, S. V. Veeravalli, Local isotropy in turbulent boundary layers at high Reynolds number. *J. Fluid Mech.* **268**, 333–372 (1994).

6. G. S. Lewis, H. L. Swinney, Velocity structure functions, scaling, and transitions in high-Reynolds-number Couette–Taylor flow. *Phys. Rev. E* **59**, 5457–5467 (1999).

7. R. Van Hout, J. Katz, Measurements of mean flow and turbulence characteristics in high-Reynolds number counter-rotating Taylor–Couette flow. *Phys. Fluids* **23**, 105102 (2011).

8. S. G. Huisman, D. Lohse, C. Sun, Statistics of turbulent fluctuations in counter-rotating Taylor–Couette flows. *Phys. Rev. E* **88**, 063001 (2013).

9. R. Ostilla-Mónico, R. Verzicco, S. Grossmann, D. Lohse, The near-wall region of highly turbulent Taylor–Couette flow. *J. Fluid Mech.* **788**, 95–117 (2016).

10. F. S. Godeferd, F. Moisy, Structure and dynamics of rotating turbulence: A review of recent experimental and numerical results. *Appl. Mech. Rev.* **67**, 030802 (2015).

11. C. Butcher, J. M. Barros, Y. Higashi, H. C.-H. Ng, T. Meuel, G. Gioia, P. Chakraborty, Okinawa institute of science and technology – Taylor–Couette (OIST-TC): A new experimental set-up to study turbulent Taylor–Couette flow. *Flow* **4**, E30 (2024).

12. D. Coles, Transition in circular couette flow. *J. Fluid Mech.* **21**, 385–425 (1965).

13. C. Van Atta, Exploratory measurements in spiral turbulence. *J. Fluid Mech.* **25**, 495–512 (1966).

14. N. D. Goldenfeld, *Lectures on Phase Transitions and the Renormalisation Group* (Addison-Wesley, 1992).

15. D. Buaria, K. R. Sreenivasan, Dissipation range of the energy spectrum in high Reynolds number turbulence. *Phys. Rev. Fluids* **5**, 092601 (2020).

16. R. T. Cerbus, C.-c. Liu, G. Gioia, P. Chakraborty, Small-scale universality in the spectral structure of transitional pipe flows. *Sci. Adv.* **6**, eaaw6256 (2020).

17. B. Eckhardt, S. Grossmann, D. Lohse, Torque scaling in turbulent Taylor–Couette flow between independently rotating cylinders. *J. Fluid Mech.* **581**, 221–250 (2007).

18. D. Lohse, K.-Q. Xia, Small-scale properties of turbulent Rayleigh-Bénard convection. *Ann. Rev. Fluid Mech.* **42**, 335–364 (2010).

19. U. Frisch, *Turbulence: The Legacy of A.N. Kolmogorov* (Cambridge Univ. Press, 1995).

20. R. Ostilla-Mónico, E. P. Van Der Poel, R. Verzicco, S. Grossmann, D. Lohse, Exploring the phase diagram of fully turbulent Taylor–Couette flow. *J. Fluid Mech.* **761**, 1–26 (2014).

21. M. Samie, N. Hutchins, I. Marusic, Revisiting end conduction effects in constant temperature hot-wire anemometry. *Exp. Fluids* **59**, 133 (2018).

22. P. M. Ligrani, P. Bradshaw, Spatial resolution and measurement of turbulence in the viscous sublayer using subminiature hot-wire probes. *Exp. Fluids* **5**, 407–417 (1987).

23. J. C. Wyngaard, Measurement of small-scale turbulence structure with hot wires. *J. Phys. E Sci. Instrum.* **1**, 1105 (1968).

24. P. Bearman, Corrections for the effect of ambient temperature drift on hot-wire measurements in incompressible flow (Tech. Rep. NTIS 197105, National Physical Lab, 1969).

25. F. E. Jørgensen, *How to Measure Turbulence with Hot-Wire Anemometers—A Practical Guide* (Dantec Dynamics, 2005).

26. H. Bruun, M. Khan, H. H. Al-Kayiem, A. Fardad, Velocity calibration relationships for hot-wire anemometry. *J. Phys. E Sci. Instrum.* **21**, 225–232 (1988).

27. G. I. Barenblatt, *Scaling, Self-Similarity, and Intermediate Asymptotics: Dimensional Analysis and Intermediate Asymptotics* (Cambridge Univ. Press, 1996).

28. A. P. Willis, The Openpipeflow Navier–Stokes solver. *Software X* **6**, 124–127 (2017).

29. G. S. Lewis, "Velocity fluctuations, wall shear stress and the transition in torque scaling at Re = 13,000 in turbulent Couette–Taylor flow," University of Texas at Austin, Austin, TX (1996).

30. H. Tennekes, J. L. Lumley, *A First Course in Turbulence* (MIT Press, 1972).

31. J. Lumley, Interpretation of time spectra measured in high-intensity shear flows. *Phys. Fluids* **8**, 1056–1062 (1965).

32. J. Wyngaard, S. Clifford, Taylor's hypothesis and high–frequency turbulence spectra. *J. Atmos. Sci.* **34**, 922–929 (1977).

33. R. Ostilla-Mónico, D. Lohse, R. Verzicco, Effect of roll number on the statistics of turbulent Taylor–Couette flow. *Phys. Rev. Fluids* **1**, 054402 (2016).

34. G. I. Taylor, Statistical theory of turbulence. *Proc. R. Soc. London Ser. A* **151**, 421–444 (1935).

35. K. Chola, P. Chakraborty, Symmetry analysis of the turbulent dissipation rate. *Phys. Rev. Fluids* **6**, L082602 (2021).

36. R. A. Antonia, H. Abe, H. Kawamura, Analogy between velocity and scalar fields in a turbulent channel flow. *J. Fluid Mech.* **628**, 241–268 (2009).

37. R. Antonia, J. Kim, L. Browne, Some characteristics of small-scale turbulence in a turbulent duct flow. *J. Fluid Mech.* **233**, 369–388 (1991).

38. S. C. Bailey, M. Hultmark, J. Schumacher, V. Yakhot, A. J. Smits, Measurement of local dissipation scales in turbulent pipe flow. *Phys. Rev. Lett.* **103**, 014502 (2009).
